# A randomized controlled trial of folic acid intervention in pregnancy highlights a putative methylation-regulated control element at *ZFP57*

**DOI:** 10.1186/s13148-019-0618-0

**Published:** 2019-02-18

**Authors:** Rachelle E. Irwin, Sara-Jayne Thursby, Miroslava Ondičová, Kristina Pentieva, Helene McNulty, Rebecca C. Richmond, Aoife Caffrey, Diane J. Lees-Murdock, Marian McLaughlin, Tony Cassidy, Matthew Suderman, Caroline L. Relton, Colum P. Walsh

**Affiliations:** 1 Genomic Medicine Research Group, School of Biomedical Sciences, Ulster University, Coleraine BT52 1SA, UK; 2 Nutrition Innovation Centre for Food and Health, School of Biomedical Sciences, Ulster University, Coleraine, UK; 3 Psychology Institute, Ulster University, Coleraine, UK; 4 MRC Integrative Epidemiology Unit, Bristol Medical School, University of Bristol, Bristol, UK

**Keywords:** Folic acid, DNA methylation, Cord blood, Offspring, Imprinting, ZFP57

## Abstract

**Background:**

Maternal blood folate concentrations during pregnancy have been previously linked with DNA methylation patterns, but this has been done predominantly through observational studies. We showed recently in an epigenetic analysis of the first randomized controlled trial (RCT) of folic acid supplementation specifically in the second and third trimesters (the EpiFASSTT trial) that methylation at some imprinted genes was altered in cord blood samples in response to treatment. Here, we report on epigenome-wide screening using the Illumina EPIC array (~ 850,000 sites) in these same samples (*n* = 86).

**Results:**

The top-ranked differentially methylated promoter region (DMR) showed a gain in methylation with folic acid (FA) and was located upstream of the imprint regulator *ZFP57*. Differences in methylation in cord blood between placebo and folic acid treatment groups at this DMR were verified using pyrosequencing. The DMR also gains methylation in maternal blood in response to FA supplementation. We also found evidence of differential methylation at this region in an independent RCT cohort, the AFAST trial. By altering methylation at this region in two model systems in vitro, we further demonstrated that it was associated with *ZFP57* transcription levels.

**Conclusions:**

These results strengthen the link between folic acid supplementation during later pregnancy and epigenetic changes and identify a novel mechanism for regulation of *ZFP57*. This trial was registered 15 May 2013 at www.isrctn.com as ISRCTN19917787.

**Electronic supplementary material:**

The online version of this article (10.1186/s13148-019-0618-0) contains supplementary material, which is available to authorized users.

## Background

Folate is an essential B vitamin required for viable embryonic and fetal development and as an important dietary constituent throughout life, fundamental in cellular biosynthesis and DNA methylation pathways [1, 2]. Folic acid (FA) is the oxidized, and more stable, synthetic form of folate which is exclusively found in supplements and fortified foods [3]. Well-established evidence from randomized controlled trials [4, 5] has led to recommendations, in place globally, that women should consume 400 μg/d FA from prior to conception until the end of the first trimester in order to protect against neural tube defects (NTDs) [6, 7]. Despite the identification of a relationship between maternal folate status and NTDs as early as 40 years ago, information on the mechanism behind the benefit of FA supplementation with respect to NTDs remains to be fully elucidated (reviewed in [8]), as does the relationship of FA, NTDs, and DNA methylation [9]. There is however little dispute in regards to the protective effect of folic acid supplementation before and in early pregnancy, which was proven in clinical trials to reduce NTDs by approximately 70% [10]. Furthermore, there remains a lack of evidence as to whether it is beneficial to mother and/or child to continue this supplementation throughout the entire pregnancy [11, 12]. FA supplementation during pregnancy has been associated with health benefits such as reduced risk of low birth weight [13], language delay [14], autism [15] and reduced risk of psychosis and other pediatric problems [16, 17]. In addition, observational studies have indicated that FA supplement use by mothers during pregnancy is associated with better cognitive health and brain development in the child [14, 18, 19], possibly related to the fact that there is a brain growth spurt at the end of the second trimester [20, 21]. However, there may also be potential adverse effects from excess folate in later pregnancy, an aspect which would also benefit from further exploration [12].

At a molecular level, there is some evidence in human that epigenetic changes could be the mechanism underpinning some of the effects of folate, both in the first trimester [8] in the prevention of NTDs, and also in the second and third trimester, as reviewed elsewhere [2]. Folate is essential for the production of S-adenosylmethionine (SAM), which provides the methyl group to the DNA methyltransferases (DNMTs), which carry out DNA methylation. DNA methylation is an essential means of maintaining transcriptional silencing at many different classes of genes when it occurs at promoter and enhancer elements, including endogenous retroviruses, genes on the inactive X, and imprinted genes [22] but can also facilitate transcription when occurring in the gene body [23–25]. DNA methylation is vital for embryonic survival and development, as mice carrying mutations in the DNA methyltransferases die in utero or shortly after birth [26, 27]. Some DNA methylation marks are inherited from the parents in the form of differential methylation on the paternal or maternal copy. This includes both the canonical imprinted loci, as well as some germline and neuronal genes [25, 28, 29], at all of which methylation plays a direct role in controlling transcription. Both animal and human studies have indicated that the fetal epigenome is vulnerable to environmental exposures, such as methyl group availability from the maternal diet [30–36].

Imprinted genes are a paradigm for the transmission of epigenetic information across generations. Methylation differences between the paternal and maternal copies of imprinted genes are established in the germ cells and are known to be important for transcriptional regulation. Accordingly, inappropriate loss or gain of methylation at imprint control regions (ICR) is a diagnostic feature for several human disorders. These regions are protected from the wave of demethylation which occurs prior to implantation by several factors, such as PGC7/STELLA [37] and ZFP57, a Krueppel-associated box (KRAB) domain zinc finger protein [38, 39]. Several studies to date have centered on analyzing the effects of nutrition in particular on imprinted genes [31–33, 40] and have shown that not only can altered diet result in an altered epigenotype, but it can also affect phenotype and predisposition to childhood and adulthood disease [41].

We have previously reported data from a randomized controlled trial of Folic Acid Supplementation in the Second and Third Trimester (The FASSTT Trial; ISRCTN19917787) where we found supplementation led to significant protection against folate depletion in mothers and offspring [42] and more recently that this led to differences in DNA methylation at some imprinted loci by using a candidate gene approach [43]. Here, we used the Infinium Methylation EPIC Beadchip Array to profile genome-wide DNA methylation levels in cord blood in an unbiased screen for regions susceptible to DNA methylation changes in response to altered FA levels. We report here that the top candidate region affected is a differentially methylated region (DMR) upstream of the gene encoding ZFP57. We verified our finding using pyrosequencing in cord blood and also show that the region responds to FA supplementation in maternal blood. Additionally, we confirm that altering methylation results in changes in *ZFP57* transcription.

## Results

### Maternal FA supplementation significantly improves folate status in mother and baby

**Fig. 1:**
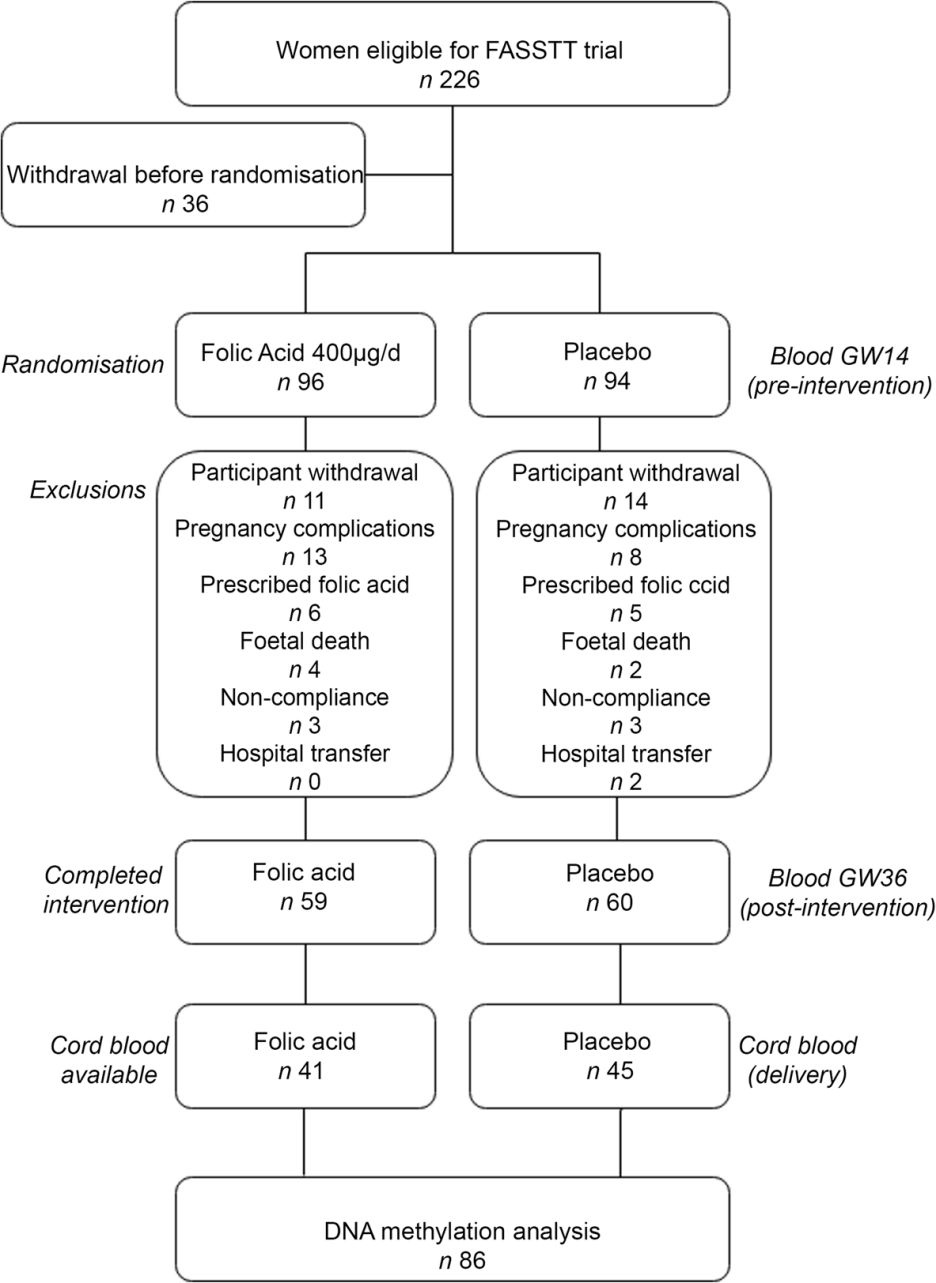
FASSTT study outline for samples used in this study. Eligible pregnant women (*n* = 226) were randomized into two groups: placebo (*n* = 94) and folic acid (*n* = 96). Women withdrew (*n* = 25) or were excluded from the intervention for the reasons indicated. A total of 119 women completed the trial. Blood samples were taken at gestational weeks (GW) 14 (pre-intervention) and 36 (post-intervention). Cord blood samples (*n =* 86) were taken at birth

For the current analysis, the same 86 cord blood samples from the FASSTT trial (outlined in Fig. 1) which had been analyzed previously for candidate gene methylation [43] were used: a summary of the most pertinent characteristics are given in Table 1 for convenience. At baseline (gestational week 14 (GW14)), there were no detectable differences between the treatment and placebo groups in maternal characteristics, dietary folate intakes, serum or red blood cell (RBC) folate concentrations, or in *MTHFR* status, as expected following randomization. There were also no significant differences in neonatal characteristics such as weight, length, and head circumference(Table 1). However, as a result of treatment with FA during trimesters 2 and 3, maternal serum and RBC folate became significantly different between placebo and treated group, as previously reported from this trial. The normal decline in maternal folate biomarkers previously reported from observational studies during pregnancy is mirrored in the placebo group where serum folate decreased from 48.8 to 23.6 nmol/L between GW14 and GW36 (Table 1). FA supplementation served to protect the mothers in the treatment group, where folate concentrations remained stable over the course of pregnancy (i.e., serum folate 45.8 nmol/L at GW14 and 46.5 nmol/L at GW36). Cord serum and RBC folate concentrations were also significantly higher in infants of the mothers supplemented with FA compared with those from the placebo mothers (Table 1). RBC folate concentrations in mothers and offspring were strongly correlated (*r* = 0.619; *p* = < 0.001, Additional file 1: Figure S1).

**Table 1:** General characteristics of participants from the EpiFASSTT trial

Characteristic	Placebo (n=45)		Folic acid (n=41)		*P* value
	*N* = 45		*N* = 41		
	Mean	SD	Mean	SD	
Maternal characteristics (GW14)					
Age (years)	28.9	3.5	29.4	3.9	0.513
BMI (kg/m^2^)	25.2	3.9	24.9	4.6	0.768
Smoker *n* (%)	8 (18)		6 (15)		0.693
Alcohol *n* (%)	3 (7)		1 (2)		0.618
Parity (*n*)	1 (1.1)		1 (1.0)		0.915
MTHFR 677TT genotype *n* (%)	5 (11)		2 (5)		0.291
Dietary intakes					
Energy (MJ/d)	8.170	1.717	7.732	1.595	0.280
Dietary folate equivalents (μg/d)	364	172	387	152	0.582
Vitamin B12 (μg/d)	4.1	1.9	3.9	1.8	0.791
Neonatal characteristics					
Gestational age (weeks)	40.1	1.3	40.0	1.1	0.540
Sex, male *n* (%)	22 (49)		22 (54)		0.659
Birth weight (g)	3610	475	3557	465	0.601
Birth length (cm)	51.5	2.6	51.1	2.2	0.499
Head circumference (cm)	34.9	1.2	34.8	1.4	0.907
Apgar score at 5 min	8.4	0.4	9.0	0.3	0.220
Caesarian *n* (%)	11 (24)		10 (24)		0.995
B-vitamin biomarkers					
Maternal pre-intervention (GW14)					
Serum folate (nmol/L)	48.8	19.8	45.8	19.5	0.469
RBC folate (nmol/L)	1185	765	1181	649	0.978
Serum B12 (pmol/L)	224	79	217	79	0.601
Maternal post-intervention (GW36)					
Serum folate (nmol/L)	23.6	17.9	46.5	24.8	< 0.001*
RBC folate (nmol/L)	991	404	1556	658	< 0.001*
Serum B12 (pmol/L)	168	51	157	60	0.229
Cord blood					
Serum folate (nmol/L)	68.3	24.8	91.7	36.7	0.004*
RBC folate (nmol/L)	1518	597	1877	701	0.024*
Serum B12 (pmol/L)	276	155	251	107	0.776

Statistical comparisons by independent *t* test (continuous variables) or *χ*^2^ test (categorical variables)

*GW* gestational week, *BMI* body mass index, *RBC* red blood cell

*p < 0.05

### Widespread alterations to DNA methylation levels in cord blood in response to late gestation maternal FA supplementation

**Fig. 2:**
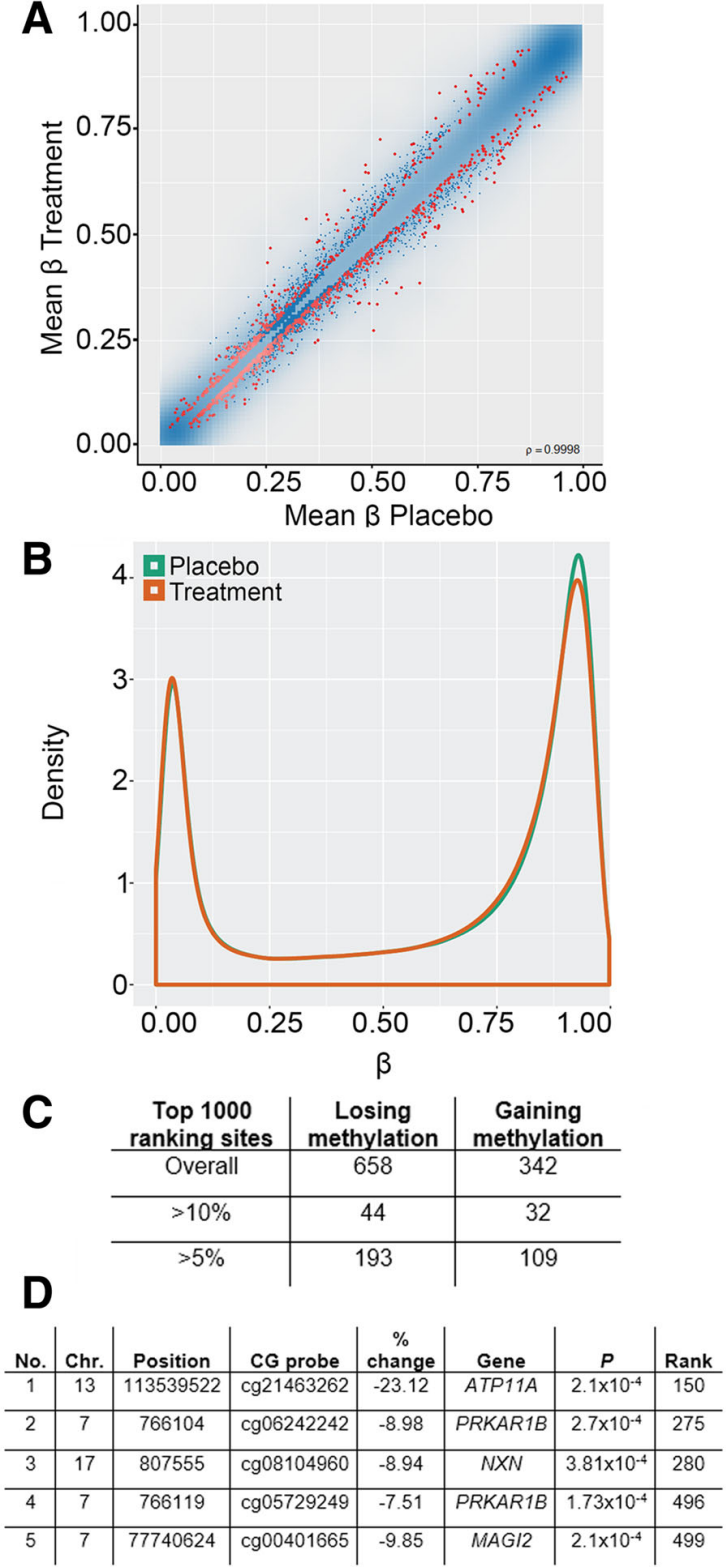
Widespread alterations to DNA methylation levels in cord blood in response to late gestation maternal folic acid supplementation. **a** Scatterplot comparing mean methylation levels (*β* values 1 = 100%; 0 = 0% methylation) at individual probes in placebo and treated groups. The 1000 top-ranking sites between groups are highlighted in red: *ρ* = correlation value. **b** Probe methylation density plot comparing the distributions of methylation values per sample group. In the treatment group, there is a decrease in the number of fully methylated sites (*β* > 0.75). **c** Split in top 1000 ranking sites losing or gaining methylation overall. Also shown are numbers of sites showing changes greater than 5% or 10%. **d** Top 5 differentially methylated sites overall, sorted by combined rank, the value being computed as the maximum (i.e., worst) value among the mean quotient log, mean difference in methylation and *p* value (*P*). No., number; Chr, chromosome; Position, coordinates in *hg19* human genome release; CG probe, identity number of the CpG probe on the EPIC array; % change, difference in mean *β* value expressed as %; Gene, nearest gene; P, probability (uncorrected); Rank, RnBeads computed ranking value (lowest being best)

DNA was purified from cord blood and quantified prior to bisulfite conversion and hybridization to the Infinium Methylation EPIC Beadchip Array, which covers more than 850,000 CpG sites distributed across the genome. Methylation values are expressed as a decimal value *β* between 0.0 (no methylation) and 1.0 (fully methylated). Data were analyzed and visualized using the RnBeads package in RStudio (see methods section). As a control, a quantile-quantile (QQ) plot of observed versus expected chi-squared values was generated and showed no evidence of population substructure effects (Additional file 2: Figure S2). Figure 2a is a scatterplot showing mean *β* value for each CpG site analyzed in treated versus placebo samples. Overall, methylation at individual CpG remains closely correlated (*ρ* = 0.998) between the two groups as expected, with most sites falling along the diagonal. Sites which differed in methylation between placebo and treatment groups were automatically ranked by RnBeads, which uses a combination of the change in mean methylation, the quotient of mean methylation and the combined *p* value, and the 1000 top-ranking sites are highlighted in red in Fig. 2a. This metric was developed to take into account not only *p* value but the magnitude of the change in methylation and in our experience is a more reliable indicator of biologically meaningful differences than *p* value alone. Sites falling along either side of the diagonal, representing gains and losses in methylation after treatment, can both be seen, with a tendency to greater numbers of sites losing. Consistent with this, a methylation density distribution plot shows that after treatment there was a clear decrease in the numbers of sites in the top quartile for methylation (*β* = 0.75–1.00; Fig. 2b). Taking the top 1000 ranking sites overall, approximately 2/3 (*n* = 658) lost and 1/3 (*n* = 342) gained methylation (Fig. 2c). However, the magnitude of these changes was generally modest, with only 302 (193 + 109) losing or gaining more than 5% methylation, the minimum change which we could potentially verify using pyrosequencing, and only 76 sites losing or gaining more than 10% (Fig. 2c).

We examined the top-ranking sites as identified by RnBeads (Fig. 2d): of these, the CpG site in the *ATP11A* gene contained a single nucleotide polymorphism (SNP) missed by the quality control routines; the same was true of the CpG at the *MAGI2* gene. The presence of the SNPs at these CpGs leads to the erroneous appearance of a change in methylation, so these were discounted. Two of the other top-ranked sites were at the *PRKAR1B* locus, which encodes a regulatory subunit of cyclic AMP-dependent protein kinase A, and one was at *NXN*, a member of the thioredoxin superfamily; however, all three were listed as located in the respective gene body and so are less likely to contribute to transcriptional control. Nevertheless, to verify these, we used a second method utilizing commercial pyrosequencing methylation assays (pyroassays) designed to query the same CpGs. These reported smaller average differences in methylation between treated and placebo groups than seen with the array of 6.6% for cg08104960 at *NXN*, and 4.2% (cg06242242) and 2.2% for (cg05729249) for the sites at *PRKAR1B*: only the site at *NXN* was significant (*p* = 0.002, *t* test).

### Identification and verification of a differentially methylated region upstream of *ZFP57*

**Fig. 3:**
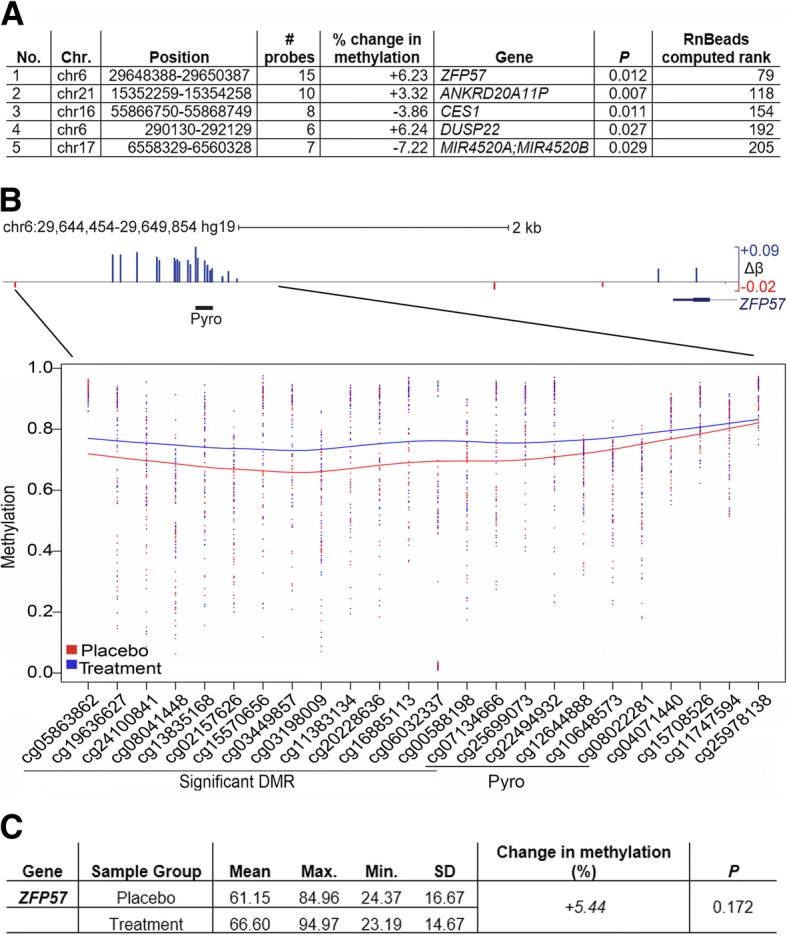
Top ranking promoter regions included imprint regulator gene *ZFP57*. **a** Top 5 differentially methylated regions (DMR) at promoters, sorted by combined RnBeads rank (smallest to largest) as for Fig. 2d above, except combining values across all the CpG sites in the DMR as detailed in the “Methods” section. Abbreviations as above except # probes, number of probes on EPIC array included in DMR. **b** Top: genome browser tracks showing the region around the DMR upstream of *ZFP57*, genomic coordinates in *hg19* human genome release, and scale as shown. EPIC array probes showing differential methylation (blue, gain; red, loss) are indicated, with size indicating the magnitude of change. The start of the *ZFP57* gene and the position of the pyrosequencing assay (Pyro) are also shown. Δ*β*, mean difference in *β* value between placebo and FA-treated groups; maximum gain and loss also shown (+ 0.09 *β* = 9% methylation). Bottom: Loess plot of *β* values across the region, with CpG identification numbers from array below; those forming the DMR defined by RnBeads are indicated, as well as sites analyzed by pyroassay. Each dot represents *β* value in an individual sample, with lines representing smoothed averages; color code is indicated at left. **c** Results of pyroassay covering the six sites indicated in **b**. Sample groups: cord blood DNA from placebo (*n* = 45) and FA-treated (*n* = 41). Mean, average of the individual means in that group; Max., largest of the mean methylation values in that group; Min, lowest mean in group; SD, standard deviation for the means; Change, difference in % methylation seen between groups; P, probability (Student’s *t* test)

Given that single sites are more susceptible to confounders such as the presence of SNPs and show only moderate accuracy on verification, and to maximize our chances of finding biologically significant changes, we also looked for genomic intervals showing coherent alterations in methylation across multiple neighboring sites [44], rather than isolated CpGs. Figure 3a lists the top 5 differentially methylated regions (DMR) found at promoters, ordered by RnBeads ranking which is here computed by combining measures at adjacent sites using a linear hierarchical model as described in the “Methods” section: uncorrected *p* value and % change in methylation are also shown for comparison. For the top 5 regions, *ZFP57* was of particular interest and is dealt with below. Two others (*CES1*, a liver carboxylesterase, and *ANKRD20A11P*, a pseudogene) showed less than 5% change in methylation and so could not be verified: *DUSP22* which has a larger change is also a pseudogene. The last DMR is located at a microRNA cluster *MIR4520A/B* and loses approximately 7.22% overall in the treatment group, averaged over a number of well-spaced CpG. Due to pyrosequencing assay design constraints, we could only cover one site (cg08750459) from the array at this locus but that site showed reasonable concordance (loss of 12.24% (*p* = 0.008) in array and 9.45% (*p* = 0.006) by pyroassay). The function of these microRNAs remains obscure however.

Of more interest in the context of this cohort was the highest ranking promoter DMR identified using RnBeads [45], which was located on chromosome 6, the closest gene being the known regulator of genomic imprinting *ZFP57*. The identified DMR consisted of 15 CpG sites and mapped approximately 3 kb upstream of the first exon of the gene, a region containing additional adjacent sites also gaining methylation. Figure 3b shows a genomic map of the first exon of *ZFP57* and the upstream region, overlaid with a track showing the locations of EPIC probes and whether they gained or lost methylation. Also shown is a graph of averaged methylation values at the numbered CpG probes from the array in placebo and treatment groups, showing a clear difference in methylation extending beyond the DMR. To confirm these results using a second method, we designed a pyrosequencing methylation assay (pyroassay) to cover some of these CpG sites, as shown in Fig. 3b. Due to the CpG density of this region, thus difficulty in pyrosequencing primer design, our pyroassay is not directly overlapping all CpGs identified by RnBeads as the DMR but is inside the area showing methylation differences. We then carried out PCR and pyrosequencing for all the samples. The overall gain in methylation at the CpGs covered by the pyroassay (*n* = 6) was very similar in magnitude and direction to that seen over the neighboring CpG by the array (+ 5.44% vs + 6.23%, respectively—Fig. 3a, c).

### Demethylation of the upstream region was accompanied by increased *ZFP57* transcription

**Fig. 4:**
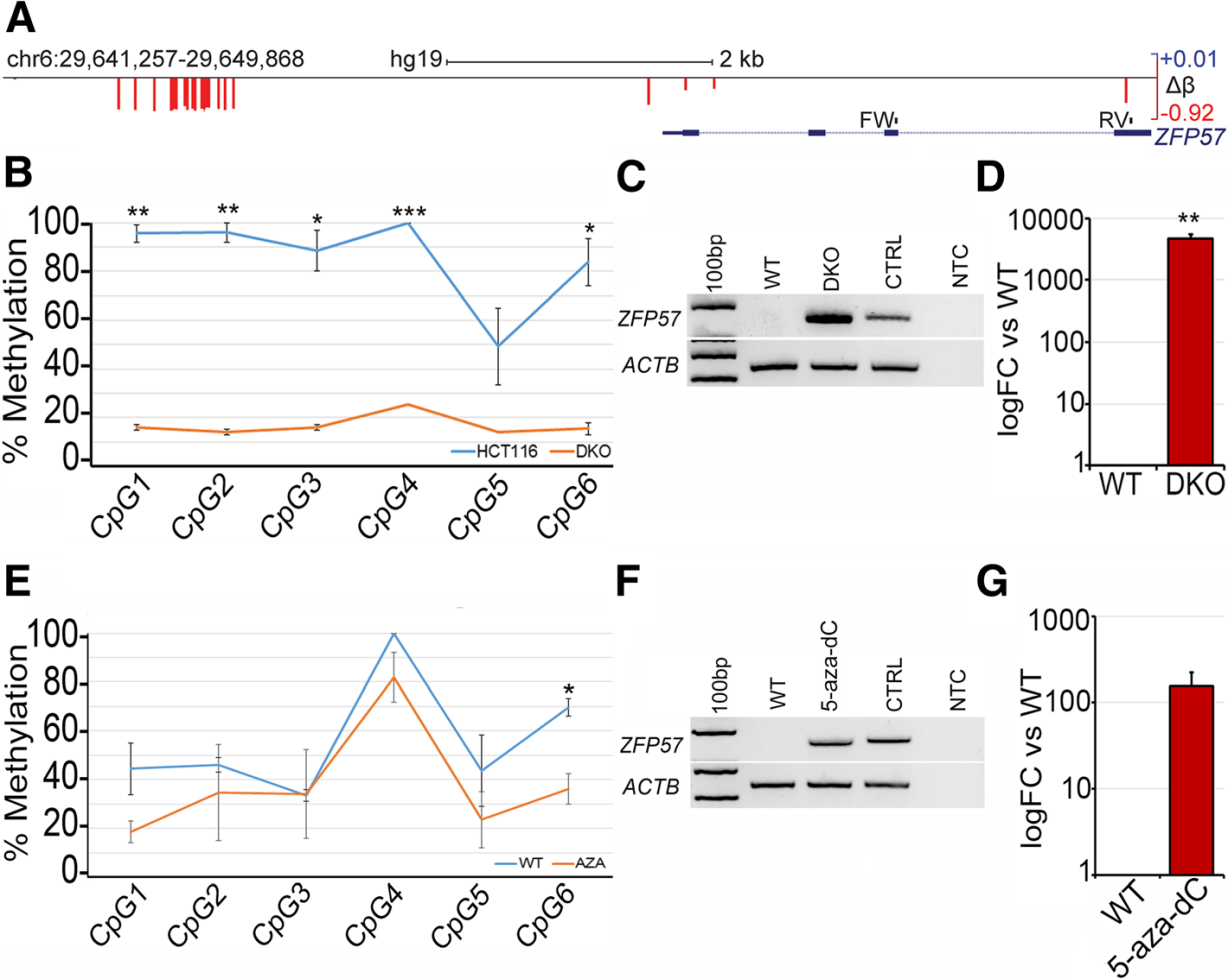
ZFP57 upstream region is a methylation-dependent regulator of transcription at this locus. **a** Schematic as in Fig. 3 above but showing difference in methylation (Δ*β*) between HCT116 WT cells vs HCT116 DKO cells. The intron/exon structure and positions of the forward (FW) and reverse (RV) primers for RT-(q)PCR on the ZFP57 gene are also shown. **b** Methylation levels at individual CpG covered by the pyrosequencing assay in WT (HCT116) and knockout (DKO) cells. Values are shown as mean +/− SD for each site: *p < 0.05; **p < 0.01; ***p < 0.001. **c** RT-PCR showing upregulation using the primers indicated in **a**, key as above. CTRL, positive control (human reference total RNA); NTC, negative control (no template control); 100 bp, size standards ladder; *ACTB*, β-actin loading control. **d** Confirmation of upregulation by RT-qPCR using the same primers, values normalized to *HPRT*; FC, fold change. **e** Methylation levels using pyroassay as in B but in 5-aza-dC treated SH-SY5Y cells (5-aza-dC), as compared to untreated (UT). **f** RT-PCR for 5-aza-dC treated cells from **e**. **g** RT-qPCR confirmation of *ZFP57* upregulation in 5-aza-dC-treated SH-SY5Y cells

Having established that methylation differences at the upstream DMR are evident between FA-supplemented and placebo-treated controls, we wished to test mechanistically if such differences could impact on transcription from the downstream gene. To do this, we first used a well-established model, the paired colorectal cancer lines HCT116 and its derivative HCT116 DKO (double knockout), which carries mutations in two of the methyltransferase genes *DNMT1* and *DNMT3B* and is known to be hypomethylated at many loci [46]. Methylation array data available in-house showed differential methylation between the parental or wild type HCT116 (WT) and paired DKO cells at the same region upstream of *ZFP57* found in the FASSTT cohort, indicated by red colored bars whose height is proportional to the loss of methylation (Fig. 4a); this indicates that DNMT1 and DNMT3B are required for methylation at this locus. We confirmed these results using our pyroassay, which showed > 80% methylation in WT HCT116 cells and a drop to < 20% in DKO cells (*p* = < 0.001) (Fig. 4b).

**Fig. 5:**
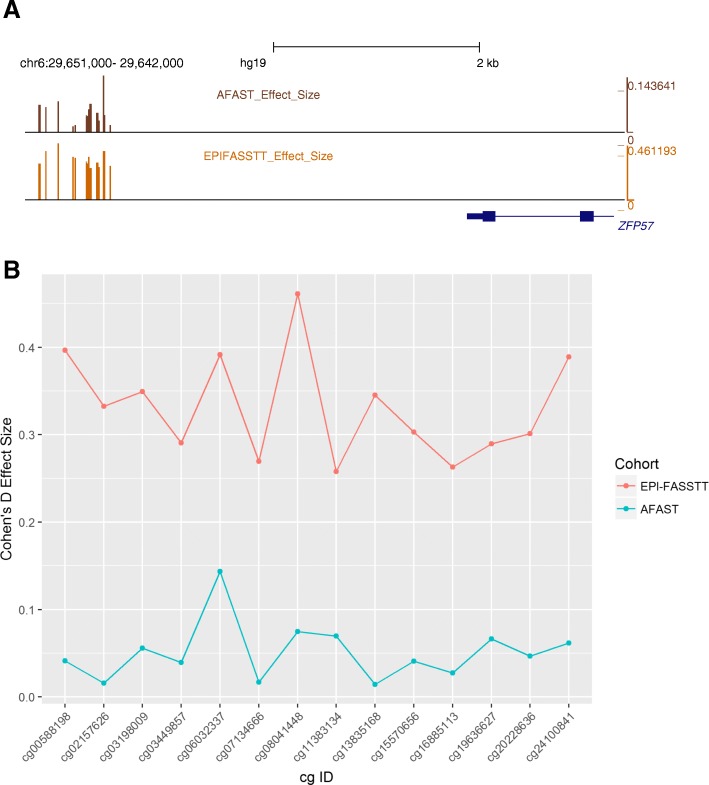
Comparison of AFAST and EpiFASSTT data for the DMR. **a** Effect size (Cohen’s *d*) at each CpG in the *ZFP57* DMR was calculated by comparing high dose and placebo from the AFAST study and plotted against the locus (top track). A similar analysis was done for the EpiFASSTT data (bottom track). Maxima are indicated at right, scale bar and location at top; note: no other CpG outside the DMR are shown in this analysis. **b** The two sets of values from **a** are plotted on the same scale to give an indication of comparability

To determine if methylation at this upstream region can regulate transcription at the *ZFP57* gene 3 kb downstream, we designed primers to cover part of the transcript as shown in Fig. 5a (FW/RV) and carried out reverse transcription on mRNA from the cells followed by polymerase chain reaction (RT-PCR). While minimal transcript could be detected in the HCT116 WT cells, which are heavily methylated, signal was readily apparent in the demethylated DKO cells (Fig. 4c). We confirmed this expression pattern quantitatively using RT-qPCR (Fig. 4d). While these results show that the gene can be de-repressed in response to loss of methylation, it is normally not expressed in colon cells, from which HCT116 were derived, so we used the neuroblastoma cell line SH-SY5Y to test the effect of methylation changes on transcription in a neural cell type. *ZFP57* is normally transcribed in neural tissue as well as early embryo [47], but shows some methylation in the SH-SY5Y cells, which may be due to differences among neural cell types, or reflect accumulation of methylation during culture; however, these cells are likelier than HCT116 to contain neural-specific transcription factors. Here, we used a second method to perturb methylation, namely treatment with the DNA methyltransferase inhibitor 5′aza-2′deoxycytidine (5-aza-dC). Exposure of the cells to this small molecule inhibitor caused loss of methylation at the upstream region (Fig. 4e). RT-PCR confirmed that *ZFP57* was de-repressed upon treatment with 5-aza-dC (Fig. 4f). Quantification of mRNA levels with RT-qPCR again indicated a substantial increase in transcription from the gene in response to loss of methylation (Fig. 4g).

### Greater variability at imprinted DMR in folate-treated samples

These results suggest that the increased methylation seen at the *ZFP57* upstream region will lead to decreased transcription. Since ZFP57 plays a role in maintaining methylation specifically at imprinted genes, we examined methylation levels at these regions using data from the EPIC array. We used germline differentially methylated regions as defined by [48] and assessed average methylation across all probes which fell within these intervals. We excluded DMR which were flagged as acquiring methylation differences somatically and also germline DMR where methylation as assessed by the array fell outside the 35–65% methylation range defined as normal in that study. This left 15 imprinted germline DMR for which the median methylation level fell within the normal range in the placebo group (Additional file 3: Figure S3A). Comparing the samples from the folate supplemented group, only the maternally imprinted neuronatin gene (*NNAT*) showed a small but significant loss of methylation in the treatment group (*p* = 0.022, Mann-Whitney *U* test (MWU)) but there was no significant difference between placebo and treatment for any other DMR. However, it was notable that 11/15 DMR showed a significantly greater variability in methylation in treated participants (*p* = < 0.001, chi-squared test), which can be seen from the greater interquartile range (IQR—see Additional file 3: Figure S3A). Along with this greater variability in the treatment group, the median methylation levels trended lower than the placebo group for almost all imprinted genes (Additional file 3: Figure S3A). We repeated this analysis using imprinted DMR as defined by Court et al. [49], which defines slightly larger DMR based on an analysis of Illumina 450 K data. After applying similar criteria as above, this left 14 DMR suitable for comparison. Using these genomic intervals, again, only *NNAT* showed a significantly different level of methylation in treated samples (*p* = 0.022, MWU; Additional file 3: Figure S3B), although *PLAG1* was also close to significant (*p* = 0.072, MWU). Again, the IQR for the imprints showed greater variability in the treated than placebo groups (*p* < 0.001, chi-squared test) and medians tended to be lower in the FA-treated group (Additional file 3: Figure S3B).

### Increased *ZPF57* methylation in response to FA in maternal blood samples

In order to investigate the effects of FA in maternal tissue, and to elucidate if this differentially methylated region upstream of *ZFP57* was directly responsive, we carried out pyrosequencing on matched maternal buffy coat samples at GW14 (*n* = 24) and GW36 (*n =* 24) (i.e., comparing the same mother’s blood sample taken before and after intervention). Pyrosequencing analysis confirmed that FA-supplemented mothers show a 5.51% increase in DNA methylation levels at this DMR after late gestation supplementation (*p* = 0.609), in contrast to non-supplemented mothers, whose methylation levels decreased 1.51% at GW36 (*p*= 0.826) (Table 2).

**Table 2:** *ZFP57* methylation in maternal blood pre- and post-intervention. DNA methylation levels of *ZFP57* DMR in maternal blood samples at GW14 and GW36.

Sample group	Gestational week (GW)	Mean methylation (%)	Standard deviation (SD)	Change in methylation (%)	*p* values
Treatment (*n* = 24)	GW14	57.47	15.37	*+ 5.51*	0.609
	GW36	62.98	14.94		
Placebo (*n* = 24)	GW14	64.36	6.58	*−1.51*	0.826
	GW36	62.85	7.13		

*GW* gestational week, *SD* standard deviation

### Effect of FA at the *ZFP57* DMR in a second cohort

In order to test the generality of the effect of folic acid intervention on this genomic region, we examined data from a second randomized-controlled trial. The Aberdeen Folic Acid Supplementation Trial (AFAST) was an RCT using two doses of folic acid (0.2 and 5 mg/day vs placebo) during pregnancy, with intervention starting at antenatal booking at < 30 weeks gestational age [50]. The study was conducted in the late 1960s, and recently, Richmond and colleagues [35] followed up on the offspring born to the mothers who had participated in the trial, mean present age of 47 years. Saliva samples were collected from those who could be identified and consented, with subsequent 450 k array analysis conducted using modeling approaches to correct for hidden variables such as cell counts [35]. Examination of the CpG in the *ZFP57* DMR which we had identified in the EpiFASSTT cohort showed the same trends in the AFAST high-folate cohort versus placebo, with change in a positive direction across the whole region (Fig. 5a), although effect size was lower at each site in the AFAST study (Fig. 5b).

## Discussion

We have previously reported DNA methylation differences at imprinted loci using cord blood from the EpiFASSTT trial of folic acid (FA) supplementation in later pregnancy by using a candidate gene approach. Here, we used the same samples to carry out an unbiased genome-wide screen for methylation differences using the EPIC array. The top hit was a differentially methylated region upstream of the imprint controller *ZFP57*, and we separately verified methylation differences by pyroassay. This region responded to FA supplementation in maternal blood as well as in cord blood and showed differences between FA-treated and untreated in an independent cohort [50]. Altered methylation at *ZFP57* was associated with increased variation in methylation at imprinted loci in cord blood. We also showed using two separate cell line models that altering methylation at the *ZFP57* upstream region can affect transcription, indicating a potential feedback mechanism may be operating here. We were also able to identify and verify methylation changes at a number of other individual CpG sites including some in the gene bodies of the *NXN* and *PRKAR1B* genes and at the start of the *MIR4520A/B* gene, but these were less likely to have functional consequences. It is notable also that we found more decreases in methylation genome-wide than increases, which may seem counter-intuitive; however, we and others have reported similar response to FA previously [43, 51]. It is has been suggested that FA may cause feedback inhibition by altering the SAM to SAH ratio and therefore the intracellular methylation potential [52].

Uncovering a DMR at a region controlling *ZFP57* transcription as the top hit in an unbiased screen was particularly striking in the EpiFASSTT randomized controlled trial, where we have already shown, using a candidate gene approach, that methylation levels were perturbed at some imprinted loci. The primary importance of ZFP57, as described in the literature from mechanistic work, is in maintaining imprinting, and it is currently the only protein known to be dedicated solely or largely to this epigenetic process [53]. ZFP57 was discovered as a maternal-zygotic effect gene which was required in mice for establishing methylation at some imprints in the oocyte, and for maintaining all imprints, both maternal and paternal, in the preimplantation embryo [38]. It does this by binding to a conserved hexamer consensus sequence (5′-TGCme5CGC-3) found at all imprinting control regions (ICRs) [54, 55], recognizing the methylated CpG in this motif, as shown in a crystallographic study [56]. Deletion of mouse *Zfp57* causes a loss of methylation from the modified parental allele by mid-gestation, with subsequent dysregulation of transcription at imprinted loci and embryonic lethality [55]. Importantly, mutations in the human homolog *ZFP57* are also associated with hypomethylation of multiple imprinted loci, indicating a conserved role in human for this gene in maintaining imprints [39].

Although this is the first report, to our knowledge, from a randomized controlled trial of FA intervention which implicates methylation changes at *ZFP57*, it was previously reported from a small observational study (*n* = 23) that maternal folate concentrations in the third trimester were associated with changes at a DMR at the same genomic location [51] when cord blood DNA methylation levels at birth were profiled. While that study reported a loss rather than gain of methylation, it was not an RCT but an observational study, and so could not test the effects of folate supplementation directly in a controlled fashion: there were many other differences in study design, numbers of participants, and analysis methods. It should also be noted that the high folate group in that study had levels of serum folate almost twice those seen in our treated samples (74.59+/− 6.1 nmol/L Amarasekera et al. vs 46.5 +/− 19.5 nmol/L GW36 treated group in this study), highlighting that we are protecting normal folate levels rather than elevating them. Although the largest-to-date observational study, comprising a meta-analysis of the MoBa (*n =* 1275) and Generation R (*n =* 713) cohorts, did not identify this region as a top hit, they could confirm that five CpG sites within this 923 bp region were significantly altered, though not the direction of change [57]. These two papers reporting changes from different observational studies nevertheless lend considerable support to this being a true folate-sensitive DMR. We could also verify using a separate biological assay the magnitude and direction of change in methylation, a gain of 5.44% in the treatment group, at the DMR in cord blood by using pyrosequencing (*p* = 0.172). Furthermore, by comparing the mother’s pre- and post-intervention, we could show that this region also gained methylation in the treated mothers, but lost methylation in the placebo group, providing a further degree of validation.

To extend our findings, we also used data from one of the few other RCTs testing the role of folic acid during pregnancy, the AFAST study [50]. We found a small effect (Cohen’s *d* < 0.2) at all the CpG across the *ZFP57* DMR, whereas there was a medium effect (Cohen’s *d* < 0.5) seen at the same region in the EpiFASSTT study. The effect in AFAST was only seen with the high dose of FA (5 mg/day) vs placebo, rather than the lower dose (200 μg/day) which was closer to that used in EpiFASSTT (400 μg/day), and the effect size was smaller than that seen in EpiFASSTT. There may be a number of reasons why effect size was smaller in AFAST: (1) the time between exposure and measurement is much greater, with median age 47 years in AFAST, vs newborns in EpiFASSTT; (2) the AFAST participants used were recruited significantly later than other groups (20.2 weeks for high dose vs 16.3 for low dose), meaning that there was less time spent exposed to the additional FA while in the womb; (3) the AFAST DNA samples were derived from saliva, while the EpiFASSTT DNA samples are from cord blood; and (4) the final numbers for the AFAST comparisons were very low (5 mg/day *n* = 23; placebo *n* = 43). Notwithstanding these limitations, the AFAST study showed a similar effect in terms of direction and magnitude at the same region upstream of *ZFP57*, providing further evidence that this is a bona fide FA sensor.

Given the role of ZFP57 in imprint maintenance, we also took advantage of the array to examine imprinted genes in our samples. Of these, only the maternal imprint *NNAT* (neuronatin) showed a small but significant loss of methylation in the treatment group, consistent with other evidence [58]. *NNAT* is highly expressed in the brain and placental tissue and functions during brain development to regulate ion channels and maintain hindbrain and pituitary segment identity [59]. *ZFP57* is essential for the maintenance of this imprint [38]. Induction of increasing mRNA levels of *NNAT* commences at midgestation in association with neurogenesis and peaks upon neuroepithelial proliferation and neuroblast formation [60], which would coincide with when folate concentrations increased in the treated group. Although we previously reported significant differences overall at *IGF2*, and at some CpG for *GRB10,* in our candidate gene approach using these samples [43], that was based on pyroassays which covered smaller regions of the imprinted DMR, whereas the probes from the array are more dispersed and cover a larger area. It was also notable that, while there was little change at other imprinted DMR as assessed by the array, there did appear to be an increase in the variability of methylation at these regions, an effect which was small but statistically significant and consistent with findings from a mouse model where FA supplementation increased variance in methylation levels across generations [61]. Given that *ZFP57* has a role in maintaining imprints, increased methylation at the upstream controller as seen in our FA-treated samples should lead to decreased transcription of *ZFP57*, which could potentially lead to reduced ability to maintain imprints and increased variability in methylation at the ICR. These possibilities can be further explored using our in vitro cell models.

It remains to be established from mechanistic studies in mouse whether ZFP57 plays any role in maintaining methylation in vivo in the post-implantation embryo. It is also possible that methylation of the DMR in human blood may not reflect the methylation levels seen at earlier stages, or in tissues which normally express the gene, which includes oocytes and some neural cells. It may be that methylation levels at the *ZFP57* DMR reported here reflect changes which have occurred in the cord and maternal bloods independently of what is occurring in the germline, and this would need to be assessed. It is also quite likely, given that imprints are thought to be established much earlier during development, that it would not be until the next generation that effects at imprinted germline DMRs could be seen. In this context, several studies have pointed to transgenerational rather than intergenerational effects at imprinted loci [62, 63]. It should also be noted that methylation levels varied substantially across the *ZFP57* DMR and between individuals (max = 94.97, min = 20.95), unlike the imprinted DMR which vary much less and may be buffered against methylation changes by multiple mechanisms.

In addition to its well-established role in imprinting, ZFP57 has also been proposed to act as a transcriptional repressor in Schwann cells, which comprise the principal glia of the peripheral nervous system [47]. Recent work from our group has indicated children born from mothers supplemented with FA in late gestation have psychosocial developmental benefits, scoring significantly higher for emotional intelligence and resilience in comparison with children not exposed to FA supplementation in later pregnancy [64]. Further work needs to be carried out to check if there are any other novel targets of ZFP57 which may be affected in later childhood and adulthood.

We sought to clarify whether an increase in methylation at the *ZFP57* DMR as seen in this RCT would have a substantial effect on the production of the protein. In order to explore whether changes in methylation can alter transcription, we utilized cell lines where the only variable was the presence or absence of DNA methylation. Our results from these two systems (HCT116 cells with methyltransferase deficiency and SH-SY5Y cells treated with an inhibitor) showed that altering methylation alone can cause changes in transcription at the *ZFP57* locus and that this is linked to changes in methylation at the DMR. Our results therefore support the hypothesis that the DMR represents an upstream control element for the gene, which we have shown from the RCT is sensitive to methyl donor status in the diet. Little is currently known about the factors controlling *ZFP57* transcription. Interestingly, the region containing the DMR does not appear to be conserved in mice and so may represent a human-specific element. However, it has features characteristic of a control element, as from examining publicly available datasets on the UCSC genome browser, there are DNAse I hypersensitive sites present here and data suggesting transcription factors may bind. We are currently exploring these aspects of the work further.

## Conclusions

Despite the limitations discussed above, we have nevertheless shown conclusively that a region upstream of the imprint controller *ZFP57* shows changes in methylation in mothers in response to intervention during later pregnancy with FA, a methyl donor, and that this effect is also evident in the cord blood in their offspring. Our findings are borne out by other observational studies as well as an independent RCT [50]. We have also clearly demonstrated that altering methylation is sufficient in itself to cause changes in transcription of the gene. These results have implications for the control of imprinting by environmental inputs and uncover a novel transcriptional control element which may be involved in this process.

## Methods

### Study design and sample collection

Samples were acquired from the FASSTT (folic acid supplementation in the second and third trimester) study cohort, a previously conducted double-blinded, randomized controlled trial in Northern Ireland described in full previously [42, 43]. To summarize in brief, women with singleton pregnancies were recruited at approximately 14 weeks of gestation from antenatal clinics at the Causeway Hospital, Coleraine (*n* = 226; Fig. 1). Women were excluded from participation if they were taking medication known to interfere with B-vitamin metabolism or if they had any vascular, renal, hepatic, or gastrointestinal disease, epilepsy, or had a previous NTD-affected pregnancy. Prior to randomization, *n =* 36 women withdrew from the study. The remaining eligible participants at the end of their first trimester were randomized into two groups; one group received 400 μg/d folic acid (*n* = 96) and the other a placebo in pill form (*n* = 94) until the end of their pregnancy. Randomization was done on a double-blind basis. Maternal non-fasting blood samples were taken at gestational week 14 (GW14), prior to intervention commencement, and at GW36, towards the end of the intervention. The study was completed by 119 women, as 71 participants were excluded during the study (see Fig. 1). A total of *n =* 37 women were excluded from the folic acid group for the following reasons: participant withdrawal *n =* 11, pregnancy complications *n =* 13, prescribed folic acid *n =* 6, fetal death *n =* 6, non-compliance *n =* 6. A total of *n =* 34 women were excluded from the placebo group for the following reasons: participant withdrawal *n =* 14, pregnancy complications *n =* 8, prescribed folic acid *n =* 5, fetal death *n =* 2, non-compliance *n =* 3, hospital transfer *n =* 2. Umbilical cord blood samples were collected after the expulsion of the placenta at delivery, along with birth weight, length, head circumference, mode of delivery, and Apgar score.

### Blood sample processing and B-vitamin biomarker determination

Blood samples were collected in EDTA-lined tubes, kept refrigerated, and processed within 4 h (excepting cord blood, processed within 24 h). Blood samples were analyzed for serum and red blood cell folate and vitamin B12 via microbiological assay as previously described [65, 66]. The buffy coat was used for methylenetetrahydrofolate reductase (*MTHFR*) 677C > T genotyping as described [67]. Quality control was affirmed by repeated analysis of stored batches of pooled samples. Intra- and inter-assay CVs were ≤ 8.2% for serum and RBC folate and ≤ 10.4% for serum vitamin B12.

### Maternal dietary analysis

Dietary data was collected using a 4d food diary in combination with a food-frequency questionnaire during the second trimester of pregnancy, with particular emphasis on a B-vitamin-fortified food intake. Dietary analysis was carried out using WISP version 3.0 (Tinuviel Software, UK) modified to segregate naturally occurring folate in foods versus folic acid fortification of foods; these were combined to enable calculation of dietary folate equivalents.

### Cell culture

HCT116 and double knockout (DKO) cells [46] were cultured in 1 g/L glucose DMEM supplemented with 10% FBS and 1× NEAA (Thermo Scientific, Loughborough, UK). SH-SY5Y cells were cultured in DMEM/F12 medium supplemented with 10% FBS (Thermo Scientific). For treatment with 5′aza-2-deoxycytidine (5-aza-dC) (Sigma-Aldrich, Dorset, UK), SH-SY5Y cells were seeded onto a 90-mm plate in complete medium, and the following day medium was replaced and supplemented with 5-aza-dC at a final concentration of 1 μM, which was renewed at 24-h intervals up to 72 h. Cells were then harvested for DNA and RNA extraction.

### Transcriptional analysis

RNA was extracted using the RNeasy Mini kit (Qiagen, Crawley, UK) according to manufacturer’s instructions. Complementary DNA (cDNA) was synthesized and RT-qPCR/RT-PCR were carried out as previously [29]. Primer sequences are listed in Additional file 4: Table S1. Human reference total RNA was used as a positive control for expression (Clontech, UK).

### DNA extraction, bisulfite conversion, and Infinium MethylationEPIC Beadchip Array

Genomic DNA was extracted from cultured cells as previously described [25] and from cord blood using the QiAMP DNA Blood Mini kit (Qiagen), according to manufacturer’s instructions. Purity and integrity of DNA were assessed by agarose gel electrophoresis and using the Nanodrop 2000 spectrophotometer (Labtech International, Ringmer, UK). DNA quantification was determined using Quant-IT PicoGreen dsDNA Assay Kit (Invitrogen, Paisley, UK). The DNA at a concentration of 50 ng/μl was sent to Cambridge Genomic Services (Cambridge, UK), who bisulfite converted the DNA in-house using the EZ DNA Methylation Kit (Zymo Research, California, USA) prior to hybridization to the Infinium Human Methylation EPIC BeadChip Array and scanning with the Illumina iScan according to manufacturer’s instructions (Illumina, Chesterford, UK).

### Bioinformatic analysis

*GenomeStudio* (Illumina v3.2) was used for initial data processing. Subsequently, *idat* files were imported into the *RnBeads* package (version 1.6.1) [45] in the freely available statistical software platform R (version 3.1.3) using the R Studio interface (Version 0.99.903). Samples were quality control checked including removal of probes with missing values, containing SNPs, or of poor quality using the *greedycut* algorithm, then sex chromosomes were removed from the analysis. Background correction was carried out using *methylumi.noob* and the methylation values of the remainder probes were normalized using *bmiq* [68]. Initial data exploration in RnBeads used principal components analysis (PCA) to explore potential correlations between the groups and known confounders such as BMI, smoking, and gender. In addition, in order to account for any hidden confounding variables in the dataset, surrogate variable analysis was carried out using the *sva* package with the Buja and Eyboglu algorithm from (1992) [69] Briefly, potential surrogate variables such as age, sample plate, Sentrix ID, and Sentrix Position were tested for association with the target variable sample group using PCA and any surrogate variable with a high correlation to sample group was adjusted for and incorporated into the making of the *limma* based linear model. The methylation intensities for each probe, each representing a CpG site, were represented as *β* values (ranging from 0, unmethylated, to 1, fully methylated), and these were plotted against genomic loci (based on *hg19*-Human Genome Build 19) using *GALAXY* software (https://usegalaxy.org/) [70] in order to visualize changes in DNA methylation on the University of California at Santa Cruz genome browser (https://genome.ucsc.edu/) as described previously [71].

### Bisulfite pyrosequencing

Primers spanning the probes of interest from the array were designed using the PyroMark Assay Design Software 2.0 and bisulfite-treated DNA PCR-amplified using the PyroMark PCR kit prior to analysis on a PyroMark Q24 according to manufacturer’s instruction (Qiagen). The primer sequences are summarized in Additional file 4: Table S1. Amplification was carried out as follows: 95 °C for 15 min, followed by 45 cycles of 95 °C for 30 s, 56 °C for 30 s, and 72 °C for 30 s, with a final elongation step at 72 °C for 10 min. Products were verified via gel electrophoresis prior to pyrosequencing analysis.

### Statistical analysis

Statistical analysis was performed using the Statistical Package for the Social Sciences software (SPSS) (Version 22.0; SPSS UK Ltd., Chertsey, UK). The results are expressed as mean ± SD, except where otherwise stated. For normalization purposes, variables were log transformed before analysis, as appropriate. Differences between treatment groups for participant characteristics were assessed using an independent *t* test for continuous variables or chi-square for categorical variables. Pyrosequencing data and RT-qPCR data were analyzed using Student’s *t* test to identify statistical differences between intervention groups. A *p* value < 0.05 was considered significant. Differential methylation analysis was conducted in *RnBeads* (see above) on a site and region level. The normalized *β* values were converted into *M* values (*M* = log2(*β*/(1-*β*)) and differential methylation between samples (placebo vs. treatment) was estimated with hierarchical linear models using *limma*. Ranking was automatically carried out in *RnBeads* and was based on the combination of the average difference in means across all sites in the promoter regions of the sample groups, the mean of quotients in mean methylation, and the combined *p* value, which was calculated from all site *p* values in the region using a generalization of Fisher’s method [72]. The smaller the combined rank for a region, the more evidence for differential methylation it exhibits.

## Additional files

Additional file 1: **Figure S1.** Correlation between folate levels in cord blood and mother. Scatterplot shows log-converted red blood cell folate (RCF) levels in nanomoles per liter (nmol/l) at gestational week 36 (GW36) for mothers (post-intervention) and matched cord blood. The line of best fit shows significant correlation between mothers and offspring (*r* = 0.619; *p* = < 0.001). (PDF 460 kb)

Additional file 2: **Figure S2.** QQ plot shows no evidence of population substructure effects. The observed Chi-squared (χ^2^) values (open circles), plotted as –log10 of the *p* value for both sample groups, fit tightly to the expected χ^2^ values (red line), indicating little evidence of association due to population substructure effects and that the top hits which deviate from the line (right-hand side) are likely to represent true differences due to loci with large effects. (PDF 332 kb)

Additional file 3: **Figure S3.** Median methylation levels at imprint control regions. Methylation levels at imprint control regions (ICR) were assessed by matching EPIC array probes to the imprint germline DMR intervals defined by [48] (A) or [49] (B) then taking the average (median) across each. The identities of each ICR and number of probes are indicated below. Boxes show the median and interquartile range for the individual averages from each group (Placebo *n* = 45, Treated *n* = 41), whiskers represent the range of values, dots indicate outliers. (PDF 1518 kb)

Additional file 4: **Table S1.** Pyrosequencing and transcriptional primer sets used in this study. Pyroassay primers are given as bisulfite converted sequence. The same primers were used for both RT-PCR and RT-qPCR. (DOCX 15 kb)

## Abbreviations

5-aza-dC: 5′aza-2′deoxycytidine
AFAST: Aberdeen Folic Acid Supplementation Trial
BBSRC: Biotechnology and Biological Sciences Research Council
BMI: Body mass index
DKO: Double knockout
DMR: Differentially methylated region
DNMT: DNA methyltransferases
ESRC: Economic and Social Research Council
FA: Folic acid
FASSTT: Folic acid supplementation in second and third trimester
GW: Gestational week
ICR: Imprint control region
IQR: Interquartile range
KRAB: Krueppel-associated box
MRC: Medical Research Council
MTHFR: Methylenetetrahydrofolate reductase
MWU: Mann-Whitney *U* test
NNAT: Neuronatin
NTD: Neural tube defects
ORECNI: Office for Research and Ethics Committees Northern Ireland
Pyroassay: Pyrosequencing methylation assay
QQ: Quantile-quantile
RCT: Randomized controlled trial
RT-PCR: Reverse transcription-polymerase chain reaction
SAM: S-Adenosylmethionine
SPSS: Statistical Package for the Social Sciences
WT: Wild type
ZFP57: Zinc finger protein 57
