# Supplementary figures and images for: A randomized controlled trial of folic acid intervention in pregnancy highlights a putative methylation-regulated control element at ZFP57
Source: Clin Epigenetics. 2019 Feb 18;11:31. doi: 10.1186/s13148-019-0618-0 (PMC6380035; doi:10.1186/s13148-019-0618-0)

Suppl.Fig.1

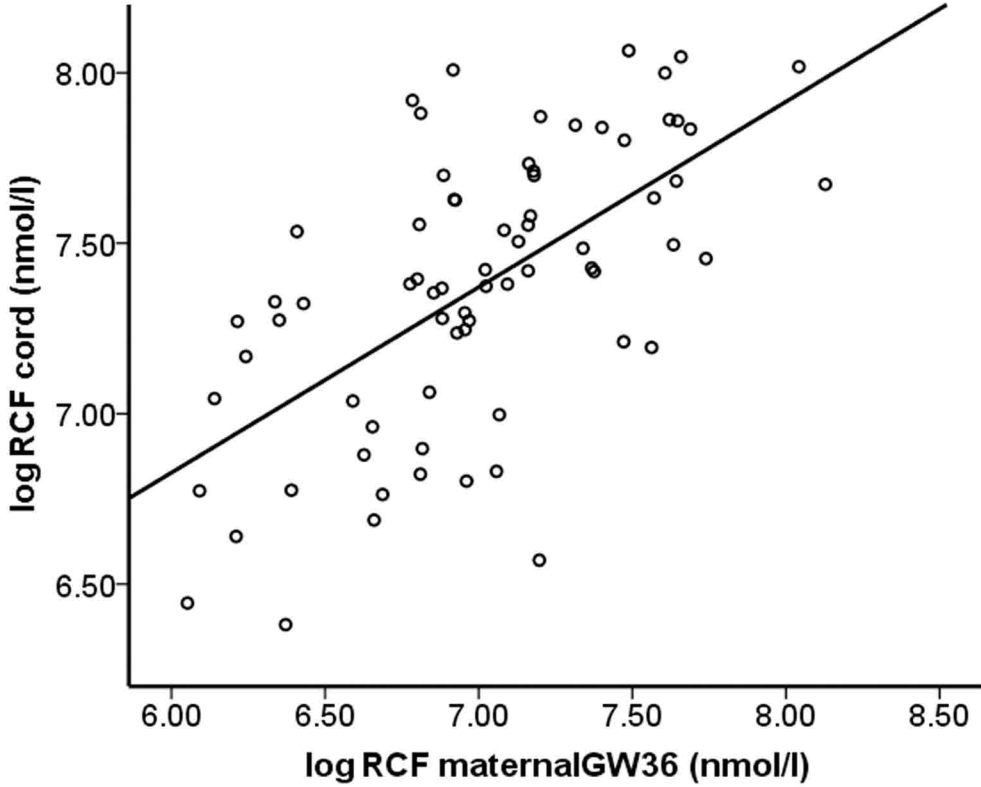

Supplement: Supplementary file 1 — Figure S1. Correlation between folate levels in cord blood and mother. Scatterplot shows log-converted red blood cell folate (RCF) levels in nanomoles per liter (nmol/l) at gestational week 36 (GW36) for mothers (post-intervention) and matched cord blood. The line of best fit shows significant correlation between mothers and offspring (r = 0.619; p = < 0.001). (PDF 460 kb) [file 13148_2019_618_MOESM1_ESM.pdf]

Suppl. Fig.2

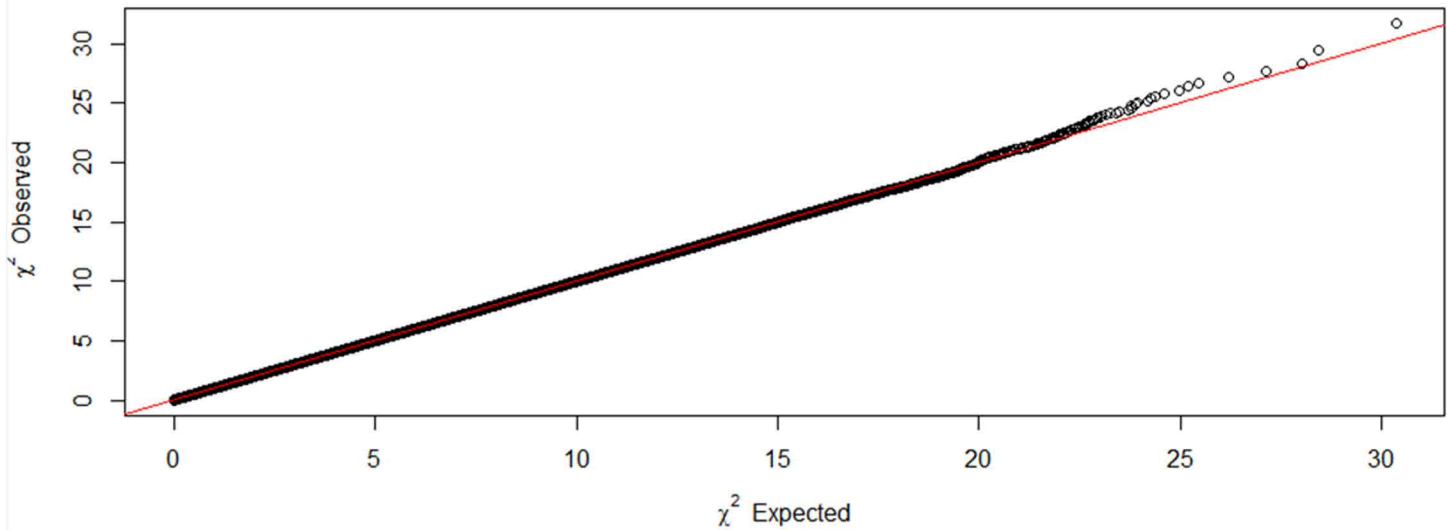

Supplement: Supplementary file 2 — Figure S2. QQ plot shows no evidence of population substructure effects. The observed Chi-squared (χ2) values (open circles), plotted as –log10 of the p value for both sample groups, fit tightly to the expected χ2 values (red line), indicating little evidence of association due to population substructure effects and that the top hits which deviate from the line (right-hand side) are likely to represent true differences due to loci with large effects. (PDF 332 kb) [file 13148_2019_618_MOESM2_ESM.pdf]

Suppl.Fig.3

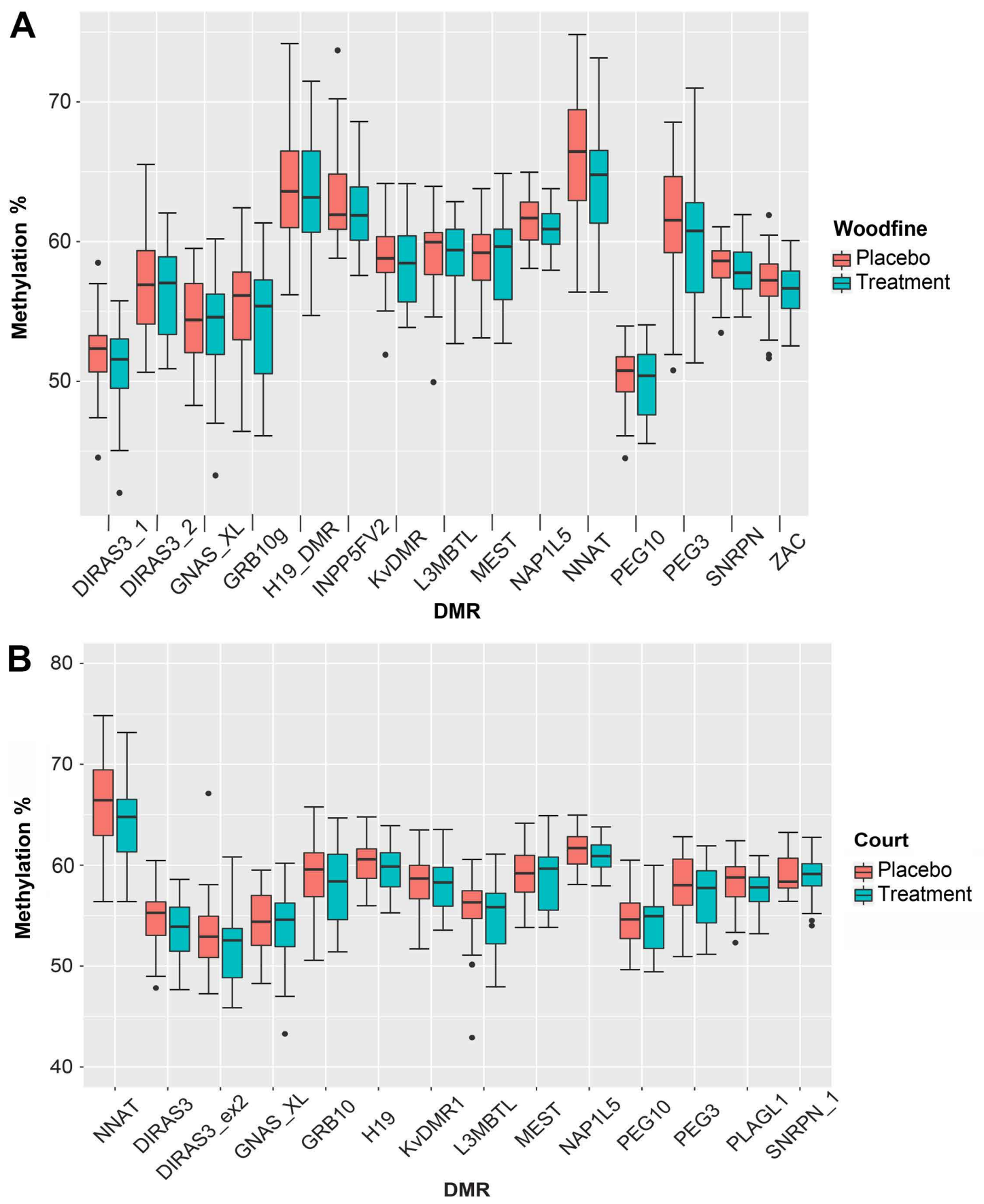

Supplement: Supplementary file 3 — Figure S3. Median methylation levels at imprint control regions. Methylation levels at imprint control regions (ICR) were assessed by matching EPIC array probes to the imprint germline DMR intervals defined by [48] (A) or [49] (B) then taking the average (median) across each. The identities of each ICR and number of probes are indicated below. Boxes show the median and interquartile range for the individual averages from each group (Placebo n = 45, Treated n = 41), whiskers represent the range of values, dots indicate outliers. (PDF 1518 kb) [file 13148_2019_618_MOESM3_ESM.pdf]
