# Supplementary material for: A randomized controlled trial of folic acid intervention in pregnancy highlights a putative methylation-regulated control element at ZFP57
Source: Clin Epigenetics. 2019 Feb 18;11:31. doi: 10.1186/s13148-019-0618-0 (PMC6380035; doi:10.1186/s13148-019-0618-0)
Supplement: Supplementary file 4 — Table S1. Pyrosequencing and transcriptional primer sets used in this study. Pyroassay primers are given as bisulfite converted sequence. The same primers were used for both RT-PCR and RT-qPCR. (DOCX 15 kb) [file 13148_2019_618_MOESM4_ESM.docx]

| **Application** | **Gene** | **Primer** | **Sequence 5’-3’** |
| --- | --- | --- | --- |
| Pyrosequencing | *ZFP57* | FW | GGGATTTTTTTTAGTTATTGTTTTGTAT |
|  |  | RV – 5’Btn | ACTAACAAACCCCTACTTTACCAAAC |
|  |  | Seq | ATTGTTTTGTATTTATTTATTAGA |
|  | *NXN* | FW – 5’Btn | TAGTAAAGTTTGGGGAAGG |
|  |  | RV | ACACCATAAAACTAAAACCAATCTAT |
|  |  | Seq | CCATAAAACTAAAACCAATCTATC |
|  | *PRKAR1B* | FW | TTTAGGGGTAGGTTTAGGTTTATAGT |
|  |  | RV – 5’Btn | CCAACCTACCTACTAAACCTTATC |
|  |  | Seq | GGTAGGTTTAGGTTTATAGTT |
|  | *MIR4520A/B* | FW | GTTTAAATTTTTTTTTGATTTGGATAGAAA |
|  |  | RV – 5’Btn | AAAACATACCCTCAATTCCAAAAAAAT C |
|  |  | Seq | TTTTTTTTGATTTGGATAGAAAATA |
| RT-qPCR/RT-PCR | *ZFP57* | FW | CCCAAACACAGAAGGCCTTT |
|  |  | RV | GGTCCTGTCCATAGTCCCAG |
|  | *ACTB* | FW | GGACTTCGAGCAAGAGATGG |
|  |  | RV | AGCACTGTGTTGGCGTACAG |
|  | *HPRT* | FW | AGCCCTGGCGTCGTGATTAGT |
|  |  | RV | CCCGTTGAGCACACAGAGGCCTA |

**Supplementary Table 1**

Primer sequences used for pyrosequencing and transcriptional analysis

Based on Human Genome Build 19; all primers listed 5’ to 3’.

Abbreviations: Btn, Biotinylated
